# Antigen-Experienced CD4lo T Cells Are Linked to Deficient Contraction of the Immune Response in Autoimmune Diabetes

**DOI:** 10.4061/2010/920148

**Published:** 2010-09-29

**Authors:** Sean Linkes, Christopher Fry, Anthony Quinn

**Affiliations:** Department of Biological Sciences, University of Toledo, 2801 W. Bancroft, Toledo, OH 43606, USA

## Abstract

Following proper activation, naïve “CD4lo” T cells differentiate into effector T cells with enhanced expression of CD4 -“CD4hi” effectors. Autoimmune diabetes-prone NOD mice display a unique set of antigen-experienced “CD4lo” T cells that persist after primary stimulation. Here, we report that a population of such cells remained after secondary and tertiary TCR stimulation and produced cytokines upon antigenic challenge. However, when NOD blasts were induced in the presence of rIL-15, the number of antigen-experienced “CD4lo” T cells was significantly reduced. Clonal contraction, mediated in part by CD95-dependent activation-induced cell death (AICD), normally regulates the accumulation of “CD4hi” effectors. Interestingly, CD95 expression was dramatically reduced on the AICD-resistant NOD “CD4lo” T cells. Thus, while autoimmune disease has often been attributed to the engagement of robust autoimmunity, we suggest that the inability to effectively contract the immune response distinguishes benign autoimmunity from progressive autoimmune diseases that are characterized by chronic T cell-mediated inflammation.

## 1. Introduction

Autoimmunity is a biological phenomenon wherein components of the adaptive immune response target host-derived molecules. Overt inflammatory autoimmune disease is a condition mediated by autoimmune mechanisms that directly or indirectly provoke tissue damage and bring about physiological consequences with clinical sequelae [1]. Surprisingly, autoimmunity may be quite common, primarily existing in a benign form with no obvious clinical manifestations [2]. On the other hand, destructive inflammatory autoimmunity is less prevalent and may be confined to a subset of individuals that share a common phenotype that predisposes them to spontaneous autoimmune disease [3–5]. Heightened and chronic autoimmune responses characterize organ-specific autoimmune diseases and may reflect poor central tolerance or an inability to delete high affinity autoreactive lymphocytes [6]. Alternatively, such clinical diseases could be a reflection of a general defect in the regulation and contraction of host immune responses that target self tissues [7]. Extensive clonal expansion of antigen-activated lymphocytes is a fundamental feature of the adaptive immune response in mammals; however, regulatory mechanisms are necessary to prevent the long-term accumulation of activated effector T cells. Following several rounds of clonal replication and expansion, the antigen-specific T cell repertoire contracts to reestablish the homeostatic levels of lymphocytes [8]. This clonal contraction is mediated in part by activation-induced cell death (AICD), a form of apoptosis that follows repeated engagements of the T cells antigen receptor [9]. AICD can be induced in vitro when recently activated T cells are restimulated through the TCR [9].

NOD mice spontaneously develop insulitis and type 1 diabetes (T1D), an autoimmune disease characterized by T cell-mediated destruction of the insulin-producing beta cells of the pancreas [1, 10]. The spontaneous appearance of cellular immune responses to islet antigens and subsequent immune-mediated pathology in the pancreas demonstrates that NOD mice lack the ability to maintain immunological homeostasis [11, 12], at least within particular organs [13]. A number of studies have provided evidence suggesting that the mechanisms normally responsible for the induction and maintenance of self-tolerance may be defective in NOD mice, rendering them unable to provide adequate protection from autoreactivity and autoimmune disease [10, 14]. NOD T cells respond abnormally to religation of the TCR, which is revealed in an initial hypoproliferative response [15, 16], a pattern of altered primary T cell division [15–18] and a failure to engage AICD upon subsequent challenge [19]. NOD mice are also deficient in the production of cytokines that can regulate T cell growth and differentiation [20, 21], which could lead to the creation of skewed T cell populations. Interestingly, a distinct subset of CD4loCD40+ T cells has been described in NOD mice [22], which may be associated with susceptibility to T1D. Contrastingly, full Th effector function may correlate with increased expression of the CD4 coreceptor, producing highly diabetogenic “CD4hi” T helper cells [23, 24]. 

Previously, we demonstrated that autoimmune-prone NOD mice display a defect in peripheral tolerance that is independent of their unique class II MHC allele (I-A^g7^) [1], but involves regulatory mechanisms such as activation-induced cell death (AICD) [19]. T cells in NOD mice may be resistant to tolerogenic signals normally delivered during thymic education [25] and those encountered in the periphery [17, 19], resulting in a loss of regulation in the specific T cell repertoires that contribute to chronic inflammation and beta cell destruction in T1D. Here we report that a subset of T cells in NOD mice is able to modulate CD4 expression and escape AICD, despite multiple rounds of antigenic stimulation. Defective cytokine-mediated maturation may lead to the accumulation of previously activated autoreactive Th cells in the periphery of NOD mice, predisposing them to chronic inflammation.

## 2. Methods

### 2.1. Mice

Four-to 8-week-old female NOD mice were purchased from Taconic Farms (Germantown NY). BALB/cJ, NOD.Scid, and NOD.BDC2.5 TCR Tg mice were purchased from The Jackson Laboratory (Bar Harbor, ME). The mice were housed in pathogen-free conditions at the University of Toledo (Toledo, OH).

### 2.2. T Cell Activation and Activation-Induced Cell Death (AICD)

Spleens were collected from naïve mice, dispersed into single cell suspensions, and treated with red blood cell lysing buffer (Sigma Aldrich, St. Louis, MO). The splenocytes were washed and resuspended in complete medium (RPMI-1640 supplemented with 10% fetal bovine serum, penicillin/streptomycin, L-glutamine, nonessential amino acids, sodium pyruvate, and *β*-mercaptoethanol) [26] with 0.5 to 2.5 *μ*g/mL of concanavalin A (Con A; Sigma) for 24–96 hours at 37°C. For secondary stimulation and the induction of AICD, Con A blasts were washed in complete medium supplemented with 10 ng/mL of recombinant human IL-2 (rIL-2), resuspended to 2.5 × 10^6^ cells/mL in medium supplemented with rIL-2, rested at 37°C for <3 hours, and then plated in triplicate on antimouse CD3*ε*-coated 96-well plates (50 *μ*l/well for 2 hrs at 37°C; clone 145-2C11; BD Biosciences, San Diego, CA). Hamster IgG-coated plates were used as controls. For tertiary stimulation, Con A blasts recovered from anti-CD3-coated 24-well plates were rested for 3 days in rIL-2 and then challenged again on anti-CD3-coated plates. 

To test proliferative responses, tritiated thymidine (1.0 *μ*Ci/well) (ICN Biomedicals Inc, Costa Mesa CA) was added to cells in 96-well plates for the last 16 hours of culture. The plates were harvested and read in a Packard TopCount NXT Microplate Scintillation and Luminescence Counter. The results were expressed as mean cpm of triplicate wells. Alternatively, the blasts were washed and placed in culture with 10 ng/mL of rIL-2 for three days to allow the “CD4lo” T cells already in transition to become “CD4hi” T cells. Afterwards, “CD4hi” and “CD4lo” T cells were separated via Ficol Histopaque (Sigma) and analyzed by flow cytometry for the expression of CD4, CD62L, and CD69. 

### 2.3. Cytokine-Induced Maturation of CD4lo T Cells

NOD splenocytes were cultured for 72 hrs in complete medium with 100 ng/mL of recombinant murine IL-2, IL-4, IL-7, or IL-15 (Peprotech, Rock Hill, NJ) plus Con A, or Con A alone. After primary stimulation the blasts were harvested and analyzed by flow cytometry for the expression of CD4. Alternatively, 72-hour Con A blasts were washed and plated in triplicate on anti-CD3-coated or IgG-coated 96-well plates for 48 hrs. One hundred microliters of SN was collected from each well and tested for the presence of IFN-*γ*. The remaining cells were pulsed with tritiated-thymidine and harvested as described above.

### 2.4. CFSE and Flow Cytometry

To monitor and enumerate the cell divisions among T cells activated with Con A or anti-CD3, spleen cells or blasts, respectively, were loaded with CFSE (Molecular Probes, Eugene, Oregon). Briefly, the cells were resuspended to 1 × 10^7^ cells/mL in RPMI, mixed with an equal volume of CFSE (20 nM), and incubated at 37°C for 5 minutes. An equal volume of cold complete medium was added to stop the reaction. Finally, the cells were pelleted and resuspended in complete medium. For primary stimulation, spleen cells were loaded with CFSE prior to plating. Con A blasts were then incubated for 96 hours, pooled, and stained with fluorochrome-labeled anti-CD4 or anti-CD8, and analyzed by flow cytometry. In some experiments, flow cytometry was performed at 24, 48, 72, and 96 hours poststimulation. To monitor cell division after secondary and tertiary stimulations, blasts were labeled with CFSE and plated on anti-CD3-coated plates and harvested at various time points** (**0, 24, 48, and 72 hours) post stimulation (secondary or tertiary). To evaluate the activation status, cells were prepared as above and stained with antibodies to CD25, CD62L, CD69, CD95, CD178, or TCR and counterstained with anti-CD4. Typically, 20,000–100,000 events were collected for flow cytometric analysis of CD4lo T cells.

### 2.5. Cytokines and ELISA

IL-2, IL-4, IL-5, IL-10, and IFN-*γ* were measured in culture supernatants at 24, 48, 72, and 96 hrs after primary stimulation and 24, 48, and 72 hrs after secondary or tertiary stimulation as previously described [27].

### 2.6. Adoptive Transfer Experiments

For adoptive transfer of T1D and/or insulitis, spleen cells from NOD.BDC2.5 TCR transgenic mice were cultured for 72 hrs in complete medium containing 2.5 *μ*g/mL of Con A. “CD4lo”-enriched cells were recovered from the blasts by underlaying the Con A blasts with Ficol Histopaque (Sigma) and centrifugation to separate the smaller “CD4lo” from the “CD4hi” blasts. After overnight culture in IL-2 containing medium—to allow complete transition of “CD4hi” cell—5 × 10^6^, Con A blasts or “CD4lo”-enriched cells were resuspended in 0.2 mL of PBS and injected into the peritoneal cavity of NOD.scid mice, a least two per group. The recipient mice were tested daily for glucosuria with Diastix (Fisher). All positives were confirmed by blood glucose measurements—levels above 250 mg/dl were considered to be indicative of T1D. For the evaluation of insulitis, the mice were euthanized 10 days after adoptive transfer. For TID, groups of 4 mice were followed for at least 30 days. Control mice received untreated spleen cells from NOD.BDC2.5 TCR transgenic mice. 

## 3. Results

### 3.1. NOD T Cells Proliferate in Response to Repeated Stimulations through the TCR

**Figure 1:**
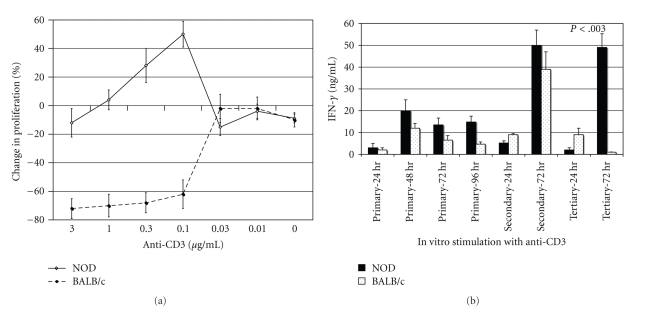
(a) Secondary NOD T cell blasts are resistant to AICD upon tertiary restimulation. NOD or BALB/c Con A blasts were restimulated on anti-CD3-coated plates, washed, and cultured in IL-2-containing medium for 3 days then stimulated again on anti-CD3-coated plates (0.003–3.0 *μ*g/mL; 50 *μ*l/well). Tritium-labeled thymidine was added for the last 16 hrs of a 72-hour culture. The data are expressed as percent change versus control wells [(mean cpm anti-CD3 wells/mean cpm IgG wells) × 100]. The standard deviation of triplicate wells was less than 10%, and the results are representative of four experiments. (b) Culture supernatants were collected at various times (24–96 hrs) following primary stimulation (Con A) of NOD or BALB/c spleen cells or secondary and tertiary stimulations of T cells blasts with plate-bound anti-CD3 (1.0 *μ*g/mL). IFN-*γ* levels were measured by ELISA, and the results are expressed as ng/mL. The results represent the mean and SD of triplicate wells and are representative of 3 independent experiments. Only *P*-values that are <.05 are shown.

To determine if defective engagement of AICD was a stable phenotype in chronically activated NOD T cells—retained through subsequent rounds of antigenic stimulation—viable NOD Con A blasts were cultured for 72 hrs on anti-CD3-coated plates (secondary), collected, and restimulated again three days later in wells coated with anti-CD3 (tertiary stimulation). Seventy-two hrs after the third round of stimulation, we observed a >40% increase in the proliferative response (Figure 1(a)) and a 10-fold increase in the secretion of IFN-*γ* among NOD T cell blasts (Figure 1(b)). Contrastingly, identical tertiary stimulation of BALB/c T cell blasts resulted in a >60% reduction in the proliferative response (Figure 1(a)) and a 90% reduction in IFN-*γ* secretion (Figure 1(b))—similar suppression of T cell activity has been attributed to AICD [9]. Thus, a detectable number of NOD T cell blasts are able to escape AICD during primary, secondary, or tertiary stimulation, and a very strong crosslinking of the TCR is needed to force NOD T cell blasts to undergo AICD, exemplified by the 3 *μ*g/mL dose in Figure 1(a).

### 3.2. NOD and BALB/c Blasts Express Similar Levels of Activation Molecules

**Figure 2:**
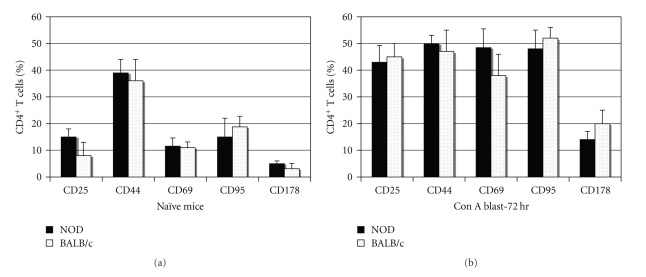
The expression of CD25, CD44, CD69, CD95, and CD178 is similar on NOD and BALB/c CD4+ spleen cells. Naive spleen cells or live Con A blasts (harvested by histopaque separation 72 hrs after stimulation) from groups of 3 NOD or BALB/c mice were examined individually by flow cytometry for the expression of cell surface molecules associated with activation. The data are expressed as the mean percentage of CD4+ cells (gated) that were positive for the respective molecule in groups of 3 mice, and the results are representative of 3 experiments, with at least 20,000 events collected in each experiment.

To demonstrate that the resistance to AICD in NOD T cells was not due to poor engagement with APC or to a lack of responsiveness to Con A, we compared the surface expression of activation molecules on splenocytes from NOD and BALB/c mice, before and after activation. There was no significant difference between the two mouse strains in the expression of CD25, CD44, CD69, CD95, or CD178 on naïve CD4^+^ splenocytes (Figure 2). Following three days of culture with Con A and recovery of viable cells, via histopaque separation, flow cytometry showed that CD4^+^ splenic blasts from NOD and BALB/c mice had increased expression of CD25, CD44, CD69, CD95, and CD178 (Figure 2). Importantly, CD95 (Fas) expression increased to over 45% in both strains, while CD178 (FasL) expression increased 3-4-fold, reaching maximal levels by 72 hours of culture (Figure 2). Overall, both strains demonstrated an ability to become engaged by Con A and upregulate surface molecules associated with activation.

Since signaling through CD95 is needed to induce AICD [28, 29], previously we evaluated the sensitivity of histopaque-sorted NOD CD4^+^ T blasts to death induced by ligation of CD95. We showed that stimulation through CD95 resulted in lost proliferative responses in NOD and control strains and a reduction in viable cell counts [19], suggesting that the CD95 pathway, including the activation of caspase 8 and 3, was functional in the NOD T cell blasts. 

### 3.3. NOD and BALB/c Mice Develop CD4hi T Cell Population during Primary Stimulation

**Figure 3:**
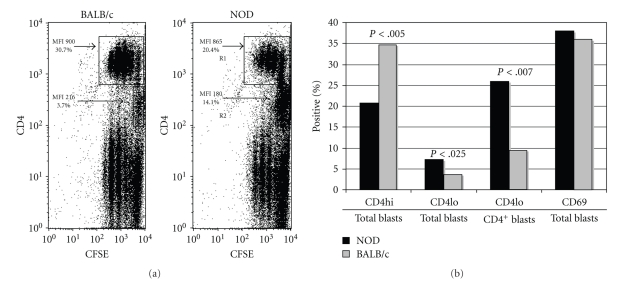
“CD4lo” T cells are increased among NOD Con A blasts. (a) Spleen cells from naïve NOD or BALB/c mice were loaded with CFSE and stimulated for 72 hrs with Con A. The resulting blasts were stained with anti-CD4-PE and analyzed by flow cytometry. The results are representative of four experiments. The data are expressed as dot plots of ungated cells (100,000 events). The numbers in the plots represent the percentage of total cells in regions R1 (CD4 MFI > 800) and R2 (CD4 MFI < 250). (b) The values in the bar graph reflect the mean percentage of cells in each category, from 3 separate experiments (100,000 events collected for each sample). Student's *t*-test was used to compare responses between NOD and BALB/c blasts (only *P*-values reaching significance, <.05, are shown).

While an overall hypoproliferative response characterizes the primary stimulation of NOD T cells [15], clearly T cells are able to expand and cause pathology in the pancreas of such mice. One of the striking features we noted in the primary response to Con A was the consistent presence of a “CD4lo” T cell population (MFI < 500) that remained among CFSE-loaded NOD splenic T cells after stimulation with Con A (Figure 3(a)). The number of “CD4lo” T cells present in NOD Con A blasts was significantly higher than that in BALB/c blasts (Figures 3(a) and 3(b)), despite the similar array of activation markers (Figures 2 and 3(b)) expressed, while the number of “CD4hi” T cells (MFI > 800) was reduced in the NOD blasts (Figure 3(b)). The typical MFI is less than 500 for freshly isolated naïve spleen cells stained with anti-CD4PE, “CD4lo” and characterizes greater than 90% of the Th cells (data not shown). Despite the disparity in “CD4lo” T cell composition, approximately 35% of the Con A blasts were CD4^+^ in both strains, suggesting that the transition to the “CD4hi” phenotype may be altered in NOD mice. Additionally, fewer cell divisions were associated with the “CD4lo” population (Figure 3(a)). 

**Figure 4:**
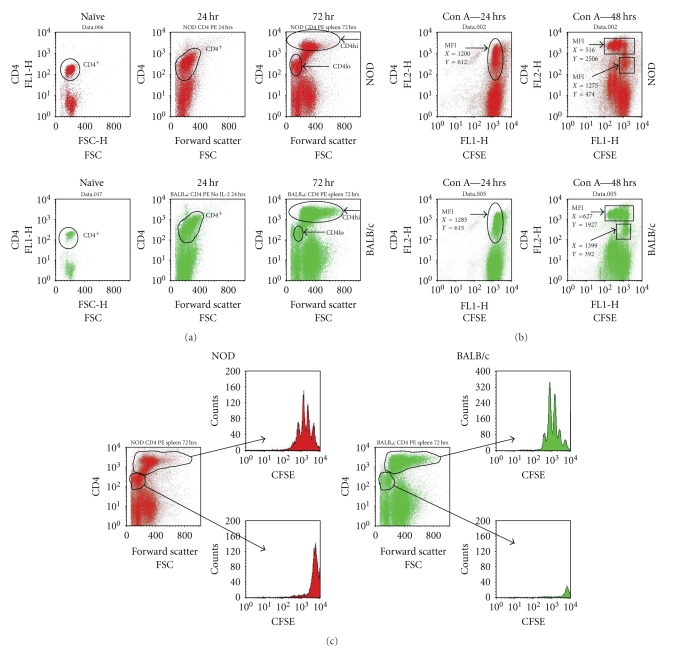
CD4 expression and T cell size increase in T cells undergoing cell division. CFSE-loaded NOD or BALB/c spleen cells were stimulated with Con A, harvested at 24 or 72 hrs, and then counterstained with anti-CD4-PE and CD3-PE/Cy5. For FACS analysis, 100,000 events were collected. The plot represents gated CD3+ cells. (a) Temporal analysis of CD4 expression and size (FSC) among Con A blasts. (b) CD4 expression and cell division (CFSE) in Con A blasts. The mean fluorescence intensity (MFI) for CFSE (*X*) and CD4 (*Y*) expression is noted for CD4+ cells at 24 and 48 hrs. (c) The pattern of CFSE expression (cell division) amongst CD4hi and CD4lo T cells from NOD or BALB/c Con A blasts 72 hrs after stimulation.

To further examine proliferation and CD4 expression among the blasts, CFSE-loaded spleen cells from NOD or BALB/c mice were analyzed directly ex vivo (naïve), or 24–72 hrs after Con A stimulation. In both strains, the naïve Th cells were all contained within a single “CD4lo” population (Figure 4(a)). However, within 24 hrs of stimulation the T cell blasts began to increase in size (FSC), as expected, and showed enhanced expression of the CD4 coreceptor (Figure 4(a)). Within 72 hours of culture, the majority of BALB/c CD4^+^ T cells had made the transition to the “CD4hi” phenotype, but we consistently observed a number of “CD4lo” T cells remaining among the NOD blasts (Figure 4(a)), demonstrating that the “CD4lo” population was detectable following activation. When we compared T cell populations from NOD, with those of BALB/c, the proliferative response for both was contained in the “CD4hi” subset, which displayed losses in CFSE intensity (MFI, *X*-axis) and concurrent increases in CD4 expression (MFI, *Y*-axis) within 48 hrs of stimulation (Figure 4(b)). Conversely, the expression of CFSE in the “CD4lo” populations was unchanged in both strains (Figures 4(b) and 4(c)). These findings confirmed that both “CD4hi” and “CD4lo” T cells were present in blasts, each with a distinct proliferation profile, such that the hypoproliferative response appeared to be associated primarily with the “CD4lo” population. We also found the “CD4lo” T cells to be smaller than the “CD4hi” when analyzed by flow cytometry (forward scatter (FSC), Figure 4(a)).

### 3.4. Hypoproliferating “CD4lo” T Cells Display Activation Markers

**Figure 5:**
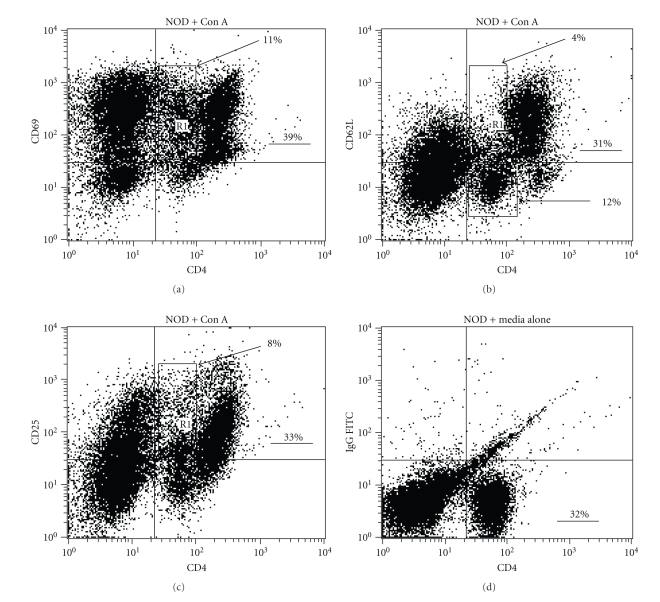
The “CD4lo” T cells that persist among NOD Con A blasts are antigen-experienced. NOD spleen cells were cultured for 72 hrs with Con A, stained with anti-CD4-PE and anti-CD69-FITC (a), anti-CD62L-FITC (b), or anti-CD25-FITC (c), and analyzed by flow cytometry (20,000 events were collected for each analysis). The selected regions (boxes) in the dot plots mark the “CD4lo” population, and the numbers indicate their percentage in the total population. The underlined numbers indicated the percentage of CD4^+^ cells in the total population. (d) Freshly isolated NOD splenocytes were stained with anti-CD4-PE and IgG-FITC for comparison to baseline CD4 expression on naïve T cells. These data are representative of two experiments.

Since hypoproliferation seem to be linked to the “CD4lo” T cells, we analyzed the expression of activation markers on the “CD4lo” T cells to further confirm that they were not virgin cells. Con A activated “CD4lo” T cells expressed levels of CD69 on their surface that were comparable to the “CD4hi” (Figure 5(a)), but retained levels of CD4 that were similar to naïve NOD spleen cultured in complete medium (Figure 5(d)). Con A activation also resulted in two-thirds of the “CD4lo” T cells losing expression of CD62L (Figure 5(b)), which is indicative of T cell activation. As expected for a fully activated T cell population, some of the “CD4hi” cells renewed the display of CD62L on their surface (Figure 5(b)). Both “CD4hi” and “CD4lo” blasts expressed CD25, although some cells from both groups displayed lower levels of the cytokine receptor (Figure 5(c)). Additionally, we found that the “CD4lo” T cells expressed CD40L at levels that rivaled or surpassed that of the “CD4hi” cells (data not shown)—the expression of CD40L has been linked to the engagement of TCR and is thus another indicator of T cell activation [30, 31].

**Figure 6:**
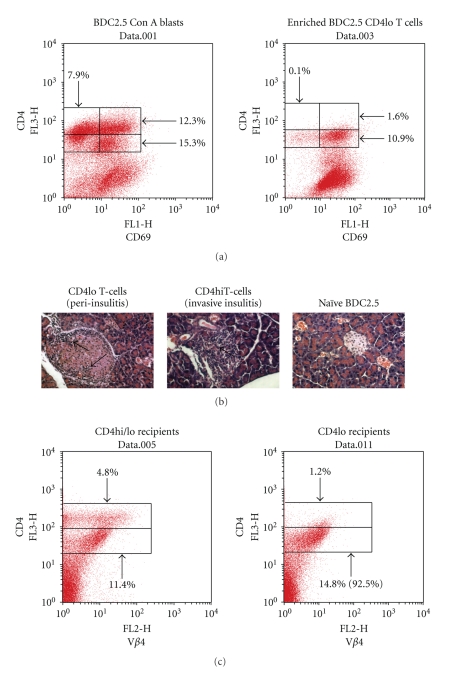
“CD4lo” T cells are present in Con A blasts produced from the spleen cells of NOD.BDC2.5 TCR transgenic mice. (a) Spleen cells from BDC2.5 TCR transgenic mice were stimulated with Con A for 72 hrs. The cells were then layered onto Ficol-Histopaque and centrifuged to recover an enriched population of the smaller “CD4lo” population. The Con A blasts and the enriched “CD4lo” population were cultured overnight in IL-2-supplemented medium and then stained with anti-CD4-PE and anti-CD69 FITC for FACS analysis. The numbers in the dot plots indicate the percentage of total cells that are within a region. A total of 20,000 events were collected for each plot. (b) 5 × 10^6^ “CD4lo”-enriched or “CD4hi”-enriched T cells were each adoptively transferred into 6 NOD.scid mice. As a control, untreated BDC2.5 TCR Tg spleen cells were also transferred into a control group of NOD.scid mice. Ten days later the pancreas was recovered from at least two mice per group, fixed, embedded, and cut into 5 micron sections that were subsequently stained with hematoxylin and eosin. The sections were viewed and photographed by a blinded observer. At least 50 islets were counted for each group to evaluate the pattern of insulitis. The arrows point to areas of inflammation, peri-insulits, which was predominant in “CD4lo” T cell recipients, and invasive insulitis, which was representative of most islets in recipients of “CD4hi” T cell recipients. The remaining mice were monitored for the development of diabetes as described in the Methods. (c) Spleen cells were recovered from the recipients in (b), stained with anti-CD4-PE and anti-TCR V beta 4-FITC, and then 100,000 events were collected during FACS analysis. The data in the dot plot were gated on viable cells (FSC × SSC). The numbers indicate the percentage of cells in the region. The number in the parentheses for the “CD4lo” recipients indicates the percentage of “CD4lo” cells in the total CD4^+^ population.

Because it was possible that the hypoproliferating “CD4lo” T cells represented a subset of NOD T cells expressing TCRs that responded poorly to Con A activation, we analyzed clonotypic blasts that were produced from the spleen cells of NOD.BDC2.5 TCR transgenic mice. We took advantage of the size difference between the two subsets and were able to enrich “CD4lo” T cells via Ficol Histopaque centrifugation—the smaller, and more dense, “CD4lo” T cells were contained in the pellet, while “CD4hi” cells were found at the interface with the separation medium. Both “CD4hi” and “CD4lo” cells were represented in the blasting clonotypic T cells, with each group expressing CD69 (Figure 6(a)). Although all of the “CD4lo” T cells expressed CD69, some of the “CD4hi” cells no longer expressed the marker, perhaps an indication of their progression to memory cells. Importantly, to determine if the “CD4loCD69+” clonotypic cells displayed true effector functions that could influence physiology, enriched “CD4loCD69+” BDC2.5 T cells, which lacked “CD4hi” T cells, were injected into NOD.Scid mice. Both “CD4hi” and “CD4lo” BDC2.5 clonotypic cells were able to transfer insulitis into NOD.scid mice (Figure 6(b)); however, only the recipients of “CD4hiCD69+” T cells developed severe insulitis (88% of the islets showed invasive inflammation) and overt diabetes (100%, 4/4) (Figure 6(b) and data not shown). Only 45% percent of the islets were severely inflamed in recipients of “CD4loCD69+” BDC2.5 blasts, while a nearly equal number displayed peri-insulitis (Figure 6(b) and data not shown). None of the mice from the “CD4lo” group (0/4) progressed to hyperglycemia. Thus, while they possess the ability to invade the target tissue, the “CD4lo” T cells were not as diabetogenic as the “CD4hi” T cells. The naive clonotypic spleen cells lacked the invasive ability (Figure 6(b)).

When spleen cells from NOD.scid recipients of “CD4lo” BDC2.5 clonotypic T cells were analyzed, only “CD4lo” T cells were detectable in the TCR V beta 4+ population (Figure 6(c)), indicating that the “CD4lo” had not differentiated into “CD4hi” T cells prior to achieving the ability to infiltrate the pancreas and induce insulitis. It should be noted that the “CD4hi” population contained both phenotypes of T cells before and after transfer.

**Figure 7:**
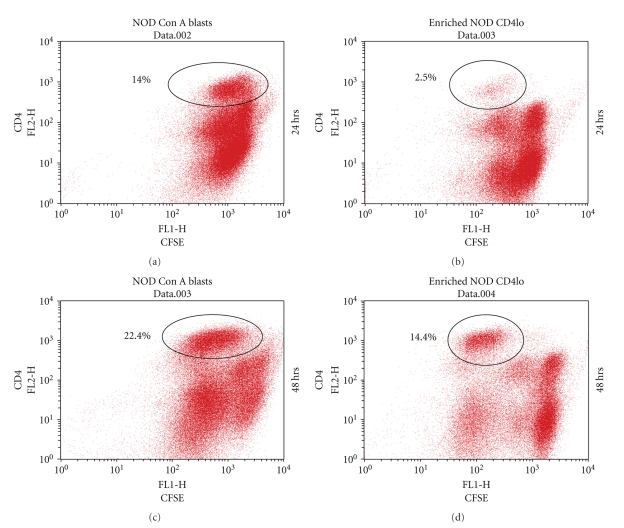
Antigen-experienced “CD4lo” T cells give rise to “CD4hi” T cells. “CD4lo” T cells were enriched from the Con A blasts of NOD spleen cells as described in Figure 6. The “CD4lo” T cells or Con A blasts were cultured overnight in IL-2-containing complete medium, then each was labeled with CFSE before incubation on anti-CD3-coated plates. Twenty-four and 48 hrs later the cells were labeled with anti-CD4-PE and analyzed by flow cytometry to reveal cell divisions. Proliferating “CD4hi” T cells arising from the blasts are indicated in the encircled population the numbers indicate the percentage in the total cell population. For each analysis, 100,000 events were collected, and the results are representative of four experiments.

To determine if the “CD4lo” phenotype among Con A blasts was preserved upon further TCR stimulation, “CD4hi” T cells were removed from NOD Con A blasts via Ficol Histopaque separation. After confirming that the remaining “CD4lo” enriched-blasts contained less than 5% “CD4hi”, the cells were labeled with CFSE and cultured on plates coated with anti-CD3 antibody. Within 24 hrs it was apparent that the “CD4lo” population had undergone at least one cell division, with many remaining “CD4lo” T cells; however, the stimulation also gave rise to a paucity of “CD4hi” T cells with reduced CFSE (Figure 7). After 48 hrs, another cell division occurred, producing a more pronounced population of “CD4hi” cells (Figure 7), thus demonstrating that “CD4loCD69+” T cells could give rise to “CD4hi” T cells. Nevertheless, a population of hypoproliferative “CD4lo” T cells still remained after the secondary challenge (Figure 7).

### 3.5. Cytokines Induce Differentiation of CD4lo T Cells

Since we were unable to correct the hypoproliferative response in the NOD “CD4lo” T cells by increasing signal strength through the TCR (increasing Con A or anti-CD3 concentrations), we investigated the role of soluble growth factors, such as cytokines, in altering the persistence of “CD4lo” T cells. Several of our preliminary experiments suggested that supernatants recovered from BALB/c Con A blasts could ameliorate the hypoproliferative response in NOD blasts when added during the primary stimulation. Therefore, to determine if specific cytokines could alter the persistence of “CD4lo” T cells, splenocytes from NOD or NOR/Lt mice were activated with Con A in the presence of recombinant mouse IL-2, IL-4, IL-7, or IL-15 and then challenged 72 hrs later on anti-CD3-coated plates. As was expected [19], the antigenic challenge of the blasts caused a reduction in the proliferative response of NOR/Lt T cell blasts (−26%) (Table 1), while inciting proliferation among the NOD blasts (+50%) (Table 1). The NOD blasts established in the presence of IL-4, IL-7, and IL-15 showed significantly enhanced proliferation; IL-15 produced the most dramatic increase (Table 2). The impact of the cytokines was less striking on NOR/Lt blasts, as none increased proliferation by more than 50%, although the response with supplemented IL-15 was significantly higher than the media control (Table 1). IL-2 was unable to enhance proliferation and may have actually enhanced cell death in blasts from both mouse strains (Table 1). The exposure to the cytokines did not appear to interrupt the blast's ability to respond to secondary antigenic challenge, as anti-CD3 was able to induce IFN-*γ* under all conditions tested (Table 1). 

To determine if enhanced proliferation would lead to alterations in the unique expression of CD4lo/CD4hi phenotype in NOD T cell blasts, we analyzed CD4 expression on Con A blasts incited in the presence of exogenous cytokine, 24 and 72 hrs after challenge with plate-bound anti-CD3. In the presence of IL-15, the percentage of “CD4hi” nearly tripled in the first 24 hrs and almost reached a fourfold increase by 72 hrs (Table 2). Similar increases were observed at 24 and 72 hrs with IL-4, while the effects of IL-2 and IL-7 were less pronounced and best seen at 72 hrs (Table 2). A decrease in the percentage of “CD4lo” was concomitant with the increase in “CD4hi” T cells (Table 2). Therefore, four cytokines that bind to receptors that share the common gamma chain were able to drive the differentiation of NOD “CD4lo” T cells into typical “CD4hi” T cells.

### 3.6. Poor Expression of CD95 on CD4lo T Cells

**Figure 8:**
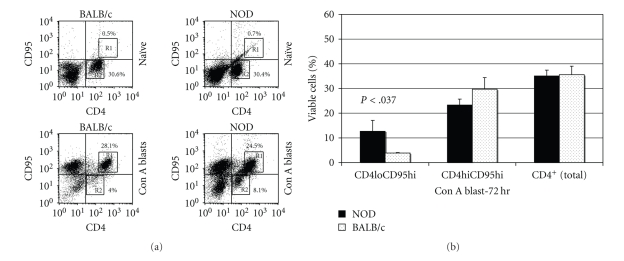
CD95 is poorly expressed on antigen-experienced CD4lo T cells. (a) Naïve and Con A activated spleen cells pooled from two NOD or BALB/c mice were stained with anti-CD4 and CD95. R1 denotes region of double positive cells, CD4hiCD95hi, in Con A blasts. R2 denotes the region of single positive cells, CD4loCD95(−). Twenty-thousand events were collected for the analysis. The numbers indicate the percentage of the total population that resides in R1 or R2. (b) A summary of 3 experiments performed as in (a), representing the mean and SD from 3 individual mice. The *P*-values are only displayed for those comparisons between NOD and BALB/c that were below  .05.

Since CD95 has been linked to AICD, we evaluated the expression of the death receptor on the two CD4 subsets. A population CD4hiCD95hi cells was highly increased following Con A activation of both BALB/c and NOD spleen cells (Figure 8(a)). However, in contrast to CD25 and CD69, the “CD95hi” T cells existed almost solely in the “CD4hi” subset, demonstrating that the transition to “CD4hi” results in an increase in CD95 expression (Figure 8(a)). The MFI for CD95 staining on the “CD4lo” cells was <127 for both groups of naïve T cells. Upon activation, the expression increased to yield distinct populations with MFIs of 394 and 311 on the “CD4hi” cells of BALB/c and NOD, respectively. On the other hand, a residual group of “CD4lo” T cells with a poor expression of CD95 (MFI = 75) remained among the NOD blasts. Similar results were observed in multiple experiments (Figure 8(b)).

## 4. Discussion

The ability to control overly aggressive immune responses may provide an important distinction between benign autoimmunity and chronic inflammatory autoimmune disease. Chronicity and dysregulation are critical aspects of autoimmunity in destructive autoimmune diseases, such that individuals who spontaneously develop autoimmune disease may be less effective in reinstating immunologic homeostasis and peripheral tolerance once autoimmunity has been initiated. Here, we investigated the relationship between T cell activation and cytokine driven T cell maturation in the NOD mouse model of autoimmunity. We found that the T cell hypoproliferative response and persistence of immunity in NOD mice were associated with “CD4lo” T cells, which appear to represent a subset that is encumbered in its progression to complete maturation and “full effector” status. The previously activated “CD4loCD69+” T cells may serve as a reservoir in the chronic autoimmunity and inflammation observed in NOD mice since such cells might be unreceptive to peripheral regulation via AICD due to poor or defective expression of the CD95 death receptor. The failure of “CD4lo” T cell blasts to progress to the “CD4hi” phenotype was corrected by the addition of cytokines that share the common gamma chain receptor, IL-4, IL-7, and IL-15 [20, 21]. Interestingly, IL-4 is associated with the prevention or delay of T1D [32–35], but the protection has typically been linked to the cytokines ability to antagonize Th-1 responses [34, 35] and regulate the proinflammatory milieu found in prediabetic NOD islets [36, 37]. Our findings suggest that the ability of IL-4 to drive T cell maturation may also contribute to its favorable impact on T1D. 

IL-4, IL-7, and IL-15 are important T cell growth and differentiation factors [20, 21]. The addition of IL-15 and IL-4 to in vitro cultures can increase the survivability of activated T cells, as can IL-7 and IL-2, with the latter two cytokines having less efficiency [38]. Here we show that the addition of IL-4, IL-7, or IL-15 at the time of activation enhances the transition of NOD “CD4loCD69+” into “CD4hi” effectors and rectifies the hypoproliferative response in the spleen. IL-4 has previously been shown to reverse the hypoproliferation in NOD thymocytes [15, 17]. It remains to be determined whether IL-15 and IL-7 can also correct the hypoproliferative response in NOD thymocytes or prevent T1D. Similar to its limited effect on T1D [32], IL-2 only partially corrected the delayed transition of “CD4loCD69+” T cells in our experiments. Additional studies are necessary to determine if IL-7 and IL-15 are qualitatively unique in NOD mice or if the expression levels are quantitatively different from that of healthy mice. Certainly there are precedents for both, as allelic variants of IL-2/IL2R may be major contributors to T1D in NOD mice [32, 39, 40]. Importantly, inadequate signaling through the common cytokine receptor *γ* chain is associated with an accumulation of T cells in the spleen [21].

Similar to the reports of others [23], we found that the expression of CD4 on Th cells from NOD and healthy controls increases following activation; however, NOD Con A blasts were unique, with a significant population of “CD4lo” remaining after multiple rounds of in vitro stimulation. This population of antigen-experienced “CD4lo” was present regardless of the concentration of Con A or anti-CD3 used to initiate the primary T cell activation. The “CD4lo” T cells were not naïve, unprimed T cells that failed to respond to TCR stimulation, as they showed increased expression of CD69 and a loss of CD62L. Furthermore, in contrasts to naïve “CD4lo” BDC2.5 T cells, clonotypic “CD4loCD69+” blasts from BDC2.5 TCR transgenic mice were able to induce insulitis in NOD.scid mice, suggesting that the adhesion molecules and chemotactic receptors necessary for extravasation were also expressed by the latter T cell population. BALB/c-derived naïve “CD4lo” T cells were much more efficient in making the transition to the “CD4hi” phenotype, with the process being 95–97% effective. Many of the CD4^+^ T cells from NOD mice made the transition to “CD4hi,” but at a much lower rate—70–75%. Paradoxically, the delayed transition into full effector status could contribute to the interval between insulitis and the onset of T1D, as some “CD4loCD69+” T cells may escape regulation, transition to “CD4hi” upon encountering antigen and later contribute to the destructive phase of insulitis—adoptive transfer experiments have shown that as few as 500 islet-derived “CD4hi” T cells can transfer T1D and only 200 are needed for tissue-specific infiltration and inflammation [24]. The activated “CD4loCD69” BDC2.5 transgenic T cells described here may have diabetogenic potential [24], but the incubation time needed after adoptive transfer may be beyond the period we tested. The extended time needed for disease transfer could be due to a need for the “CD4lo” T cells to upregulate CD4 and become fully functional effectors. The precise role of “CD4lo” T cells in T1D is unclear, as some have observed “CD4loCD40+” T cells to be highly diabetogenic [22], while others report that the “CD4hi” phenotypes are a characteristic of the most diabetogenic T cells [24]. Our findings were more consistent with the latter, as “CD4hi” T cells from BDC2.5 TCR Tg mice were more efficient at inducing T1D upon adoptive transfer. It should be noted that the “CD4hi” T cells were best observed when the anti-CD4 antibody was conjugated to PE.

While extensive clonal expansion of activated lymphocytes is a fundamental feature of the adaptive immune response, mechanisms exist to prevent the chronic accumulation of such activated T cells. Activation-induced cell death (AICD) contributes significantly to the contraction of the activated lymphocyte pool [28, 29]. Following antigen-exposure, T cells differentiate into distinct populations that vary in susceptibility to AICD upon engagement of their antigen receptors: naïve and long-lived memory T cells are much more resistant to AICD than effector T cells [41–44]. We found that NOD “CD4hi” T cells expressed CD95 and were very susceptible to AICD induced by the crosslinking of CD95, confirming that the CD95 pathway, including the downstream effector molecules were functional in NOD “CD4hi” T cells (data not shown). Contrastingly, the NOD “CD4loCD69+” T cells appear to represent a population that is able to modulate its sensitivity to AICD by altering the expression of receptors/ligands like CD95.

It is evident that naive CD4^+^ T cells encounter antigen in a state where the total surface expression of CD4 is reduced, compared to recently activated blasts; however, the connection between TCR signaling and CD4 expression is not completely clear. The TCR signaling cascade is essential for activation, differentiation, and AICD, and several kinases are influential in TCR signaling, including Lck and ZAP-70 [45, 46]. The recruitment of Lck correlates with increased AICD in mature cycling T cells [47, 48], providing supportive evidence for Lck activity leading directly or indirectly to the upregulation of CD95 on the cell surface. The expression or functional activity of Lck may be defective in NOD mice—Lck is recruited to the TCR less frequently in activated NOD thymocytes when compared to a healthy control strain [18]. As a result of decreased Lck frequency, a disproportionate number of NOD CD4^+^ T cells may not experience full activation and subsequent effector function as demonstrated by the residual “CD4lo” population. In addition, Lck recruitment to the T signaling scaffold also impacts activation of ZAP-70, costimulation, and the MAPK kinase pathways [49, 50], which can effect cytokine production. The decreased Lck kinase activity in NOD mice may also have an indirect impact on AICD by compromising T cell secretion of cytokines needed for reaching a critical threshold of divisions, and susceptibility to AICD [8].

## 5. Conclusion

Poor thymic selection via unique MHC molecules likely contributes significantly to the selection of autoreactive T cells. However, a compromised ability of such T cells to fully differentiate and engage the proper regulatory mechanisms in at-risk individuals may also contribute significantly to the chronic inflammation and postadolescent onset that characterizes many autoimmune diseases. Poor peripheral regulation could result in the formation of a repository of autoreactive T cells that have a threshold for activation lower than that of naïve cells-*γ* c-deficient mice show an age-dependent accumulation of activated T cells in the spleen and CD4−/− T cells have been shown to survive repeated antigenic challenge and activation-induced apoptosis [51]. Chronic inflammation could arise in mice where T cells poorly express CD4, but have specificity for target antigens that persist in vivo. This chronic response may not be observed with foreign antigens since the stimulating epitope would be lost once the antigen depot is cleared—barring molecular mimicry. Thus, new considerations must be exercised when developing therapeutics since correcting immune dysregulation in autoimmune-prone individuals may be more challenging than that observed in normal healthy individuals, such that paradigms for tolerance induction developed in healthy mice may not be directly applicable to autoimmune-prone individuals.

## Figures and Tables

**Table 1:** The addition of cytokines to the primary stimulation increases the proliferative response in NOD Con A blasts upon secondary challenge with anti-CD3.

Conditions of primary blasts stimulation^a^	Secondary proliferation^b^	Stimulation index^c^	IFN*γ* absorbance^d^
NOD			
Con A (no 2^0^)	22		0.24
Con A	33*	1.50**	1.13
Con A+IL-2	30	1.36**	1.01
Con A+IL-4	42*	1.91	1.21
Con A+IL-7	60*	2.72**	1.31
Con A+IL-15	90*	4.09**	1.51

NOR			
Con A (no 2^0^)	38		0.30
Con A	28*	0.74	1.54
Con A+IL-2	23*	0.61	1.35
Con A+IL-4	42	1.11	1.42
Con A+IL-7	42	1.11	1.38
Con A+IL-15	52*	1.37	1.65

^a^Splenocytes were cultured for 72 hrs with 2.0 *μ*g/mL of Con A, with or without recombinant mouse IL-2, IL-4, IL-7, or IL-15.

^b^Con A blasts were plated in triplicate on IgG-coated (no 2^0^) or anti-CD3-coated plates for 48 hrs, pulsed with H^3^-thymidine for an additional 18 hrs, harvested, and read in a beta counter. The results are the means, rounded to the nearest 10^4^ CPM. The SD was less than 10%. The asterisk (*) indicates *P* < .05, when compared to proliferation on IgG coated plate.

^c^The stimulation index (SI) was calculated as mean experimental (anti-CD3 plate) CPM/ mean Con A control (IgG plate) CPM. The two asterisks (**) indicate that *P* < .05 when the NOD SI was compared to the SI of NOR blasts treated under the same conditions.

^d^Supernatants collected 48 hrs after culture on IgG or anti-CD3-coated plates were tested by ELISA. The results are expressed as mean absorbance (405 nm) of duplicate wells. The absorbance values (1.0–1.54) represent an IFN-*γ* concentration range of 1.5–2.5 ng/mL.

**Table 2:** The addition of cytokines during primary stimulation increases the transition of naïve NOD T cells to CD4hi effectors.

Primary stimulation^a^	24 hr^b^		72 hr^b^	
	CD4lo^c^	CD4hi	CD4lo	CD4hi
Con A	58%	10	50	12
Con A+IL-2	42*	14	25*	21*
Con A+IL-4	42*	25*	14*	39*
Con A+IL-7	42*	15	22*	28*
Con A+IL-15	40*	30*	19*	40*

^a^ Splenocytes were cultured in 24-well plates for 72 hrs with 2.0 *μ*g/mL of Con A, with or without recombinant mouse IL-2, IL-4, IL-7, or IL-15.

^b^ Twenty-four or 72 hrs after the addition of Con A, the cells were collected and stained with anti-CD4-PE and analyzed by flow cytometry. The data are expressed as mean percentage of “CD4lo” and “CD4hi” cells in triplicate wells, amongst the total CD4+ population. The SD was less than 20%. The asterisk (*) indicates that *P* < .05 when compared to Con A blasts induced without cytokines added. The results are representative of two experiments.
